# Supplementary material for: The Link between Autosomal Dominant Polycystic Kidney Disease and Chromosomal Instability: Exploring the Relationship
Source: Int J Mol Sci. 2024 Mar 2;25(5):2936. doi: 10.3390/ijms25052936 (PMC10932443; doi:10.3390/ijms25052936)
Supplement: Supplementary file 1 [file ijms-25-02936-s001.zip › ijms-2872696-supplementary.pdf]

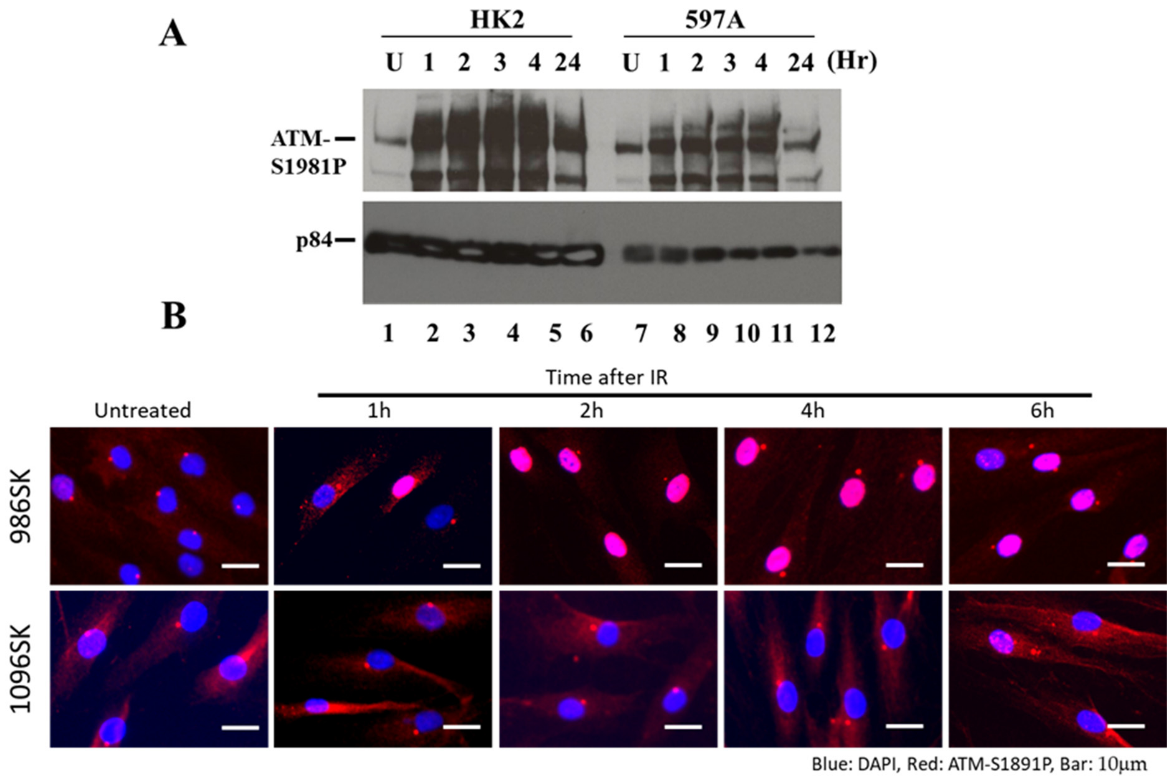

Figure S1:
DNA Damage Response in ADPKD Cells: (A) Western Blotting analysis revealing phosphorylation-dependent activation of ATM in primary untransformed ADPKD cells (597A). (B) Delayed Checkpoint Activation in Primary ADPKD Cells: Skin fibroblast cells, 986Sk (normal) and 1096Sk (ADPKD), were either left untreated or exposed to γ-radiation (IR, 10 Gy). Cells were fixed at the indicated time points and immunostained with primary antibodies against phosphorylated ATM-S1981 and phosphorylated CHK2-T68. Representative microscopic fields are depicted.

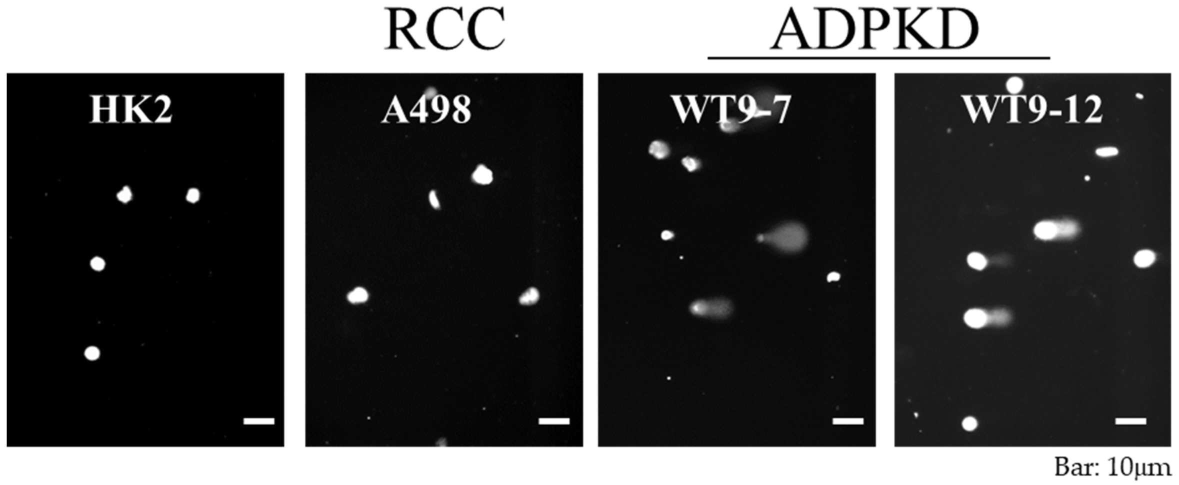

Figure S2:
Detecting Excessive DNA Damage in ADPKD Cells Using Comet Assay. Presented here are illustrative instances of individual cells undergoing alkaline gel electrophoresis and staining with SYBR Green. This process allows visualization of fast-migrating, damaged DNA in the characteristic "comet" tails (Comet Assay™) [15,16].
